# Molecular insights into proton coupled peptide transport in the PTR family of oligopeptide transporters^☆^

**DOI:** 10.1016/j.bbagen.2014.05.011

**Published:** 2015-03

**Authors:** Simon Newstead

**Affiliations:** Department of Biochemistry, University of Oxford, Oxford OX1 3QU, UK

**Keywords:** Major facilitator superfamily, Peptide transport, PTR/NRT1/POT family, Drug transport

## Abstract

**Background:**

Cellular uptake of small peptides is an important physiological process mediated by the PTR family of proton-coupled peptide transporters. In bacteria peptides can be used as a source of amino acids and nitrogen. Similarly in humans peptide transport is the principle route for the uptake and retention of dietary protein in the form of short di- and tri-peptides for cellular metabolism.

**Scope of the review:**

Recent crystal structures of bacterial PTR family transporters, combined with biochemical studies of transport have revealed key molecular details underpinning ligand promiscuity and the mechanism of proton-coupled transport within the family.

**Major conclusions:**

Pairs of salt bridge interactions between transmembrane helices work in tandem to orchestrate alternating access transport within the PTR family. Key roles for residues conserved between bacterial and eukaryotic homologues suggest a conserved mechanism of peptide recognition and transport that in some cases has been subtly modified in individual species.

**General significance:**

Physiological studies on PepT1 and PepT2, the mammalian members of this family, have identified these transporters as being responsible for the uptake of many pharmaceutically important drug molecules, including antibiotics and antiviral medications and demonstrated their promiscuity can be used for improving the oral bioavailability of poorly absorbed compounds. The insights gained from recent structural studies combined with previous physiological and biochemical analyses are rapidly advancing our understanding of this medically important transporter superfamily. This article is part of a Special Issue entitled Structural biochemistry and biophysics of membrane proteins.

## Highlights

• Crystal structures of PTR family transporters
• Identification of mechanistically important salt bridge interactions.
• Conservation of key functional residues between bacterial and mammalian homologues.
• High resolution structural information on peptide binding.

## Graphical abstract

**Figure:**
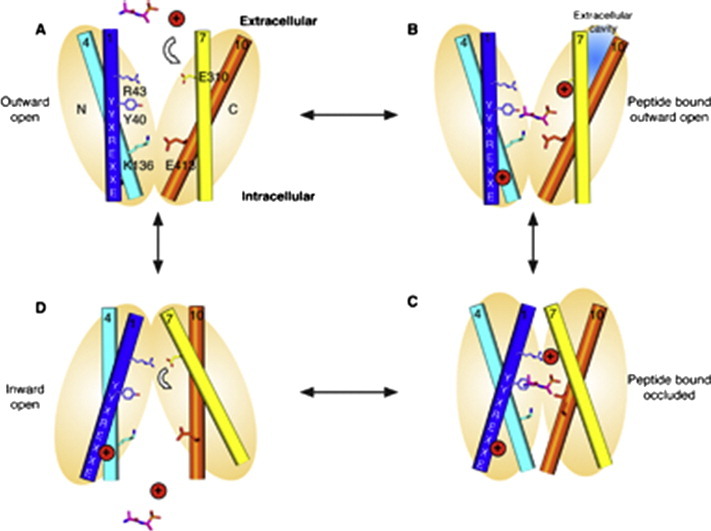

## 1 Main article

Peptide transport is one of the main routes through which cells obtain nitrogen and amino acids for metabolism and growth [1,2]. In bacteria at least three different systems have been identified that actively uptake peptides from the periplasm across the inner membrane into the cytoplasm. These include the Opp and Dpp systems that belong to the ATP Binding Cassette (ABC) transporter family [3,4], the oligopeptide transporters [5] and the peptide transporter or PTR family [6]. This review focuses on the PTR family (pfam00854), which in contrast to the Opp and Dpp systems are members of the Major Facilitator Superfamily (MFS) of secondary active transporters and are also referred to as the POT or proton dependent oligopeptide transporter family [7]. Unlike the other peptide transport systems the PTR family are the only members to have homologues in mammalian genomes [8]. Remarkably PTR members in plants, which are clustered with the nitrate transporter 1, or NRT1 family, have evolved to recognize and transport other nitrogenous ligands including nitrate, the plant hormone auxin and glucosinolate compounds used for seed defense against insect predation [9].

Although the Opp, Dpp and PTR systems are structurally and functionally distinct, they do all share one common feature — substrate promiscuity. Any system whose function is to transport peptides from the environment for nutritional assimilation should, by necessity not be too specific for certain sequences so as to avoid restricting uptake to only a subset of the available peptides. Unsurprisingly, early studies on the periplasmic binding proteins from the Opp ABC transporters discovered that these proteins could indeed recognize a diverse range of peptides [10]. This promiscuity is also observed in the PTR family [11]. However, there exists a key functional difference between these two systems. Whereas in the Opp system transport is driven by the hydrolysis of ATP, essentially decoupling peptide recognition from the driving force for transport, within the PTR family peptide binding and recognition are intimately coupled with proton binding and transport. The molecular basis for how a single binding site can be both highly promiscuous with respect to peptide recognition, while retaining a conserved proton coupling mechanism is one of the key questions in this field of research with important implications for understanding the nature of binding promiscuity in nutrient transporters.

In humans peptide uptake via the PTR transporters PepT1 and PepT2 is the main route through which the body absorbs and retains dietary protein [8,12,13]. Ingested protein is broken down into peptide fragments and free amino acids through acid hydrolysis in the stomach and the action of non-specific peptidases in the small intestine. The resultant peptides are then actively transported across the intestinal brush border membrane through PepT1 [14–18]. Peptide transport also occurs at the renal epithelium in the kidney, where PepT2, a functionally related paralogue of PepT1 reabsorbs peptides from the glomerular filtrate [19,20]. It is estimated that PepT1 and PepT2 can recognize and transport > 7000 different combinations of di- and tri-peptides, making them some of the most promiscuous transporters in biology [1,21,22].

The first PepT1 gene was cloned from a rabbit cDNA library in 1994 [15]. The primary structure of PepT1 and PepT2 predicted 12 transmembrane (TM) spanning alpha helices [14]. PepT1 and PepT2 exhibit different kinetic properties; PepT1 is a high capacity, broad specificity transporter with K_M_ values in the low mM range for most di-and tripeptides [17] whereas PepT2 exhibits higher affinity for peptides with K_M_ values in low μM range and also appears to be more selective with respect to side chain accommodation [19]. This difference has been postulated to facilitate the role these two proteins play, with PepT1 being responsible for rapid peptide uptake in the small intestine following food ingestion, whereas PepT2 must be more efficient in peptide recognition to avoid loss of these nutrients in the urine.

In addition to dietary peptide absorption, PepT1 and PepT2 also recognize several important families of drug compounds that exhibit a steric resemblance to peptides. These include the commonly prescribed β-lactam antibiotics cefadroxil and cefalexin [23–25]. In recent years significant attention has been focused on the promiscuity of both PepT1 and PepT2 as drug delivery vehicles to improve the uptake of poorly absorbed or retained medications [26]. In vivo studies in mice have shown convincing evidence that PepT1 and PepT2 substantially improve the bioavailability of molecules that have been modified through the attachment of either a single amino acid or di-peptide to create so called peptide pro-drugs [27,28]. These modified drug molecules exhibit more favorable oral bioavailability profiles and may represent an important tool in the development of more effective medications [29–32]. However a detailed understanding of the mechanism by which peptide transporters both recognize and transport natural peptides and their related drug molecules is currently absent and one of the major intellectual driving forces for their continued study [33–38].

PTR family transporters belong to the Major Facilitator Superfamily (MFS) [39] and are proton (H^+^) driven symporters, using the inwardly direct proton electrochemical gradient (Δμ_H_^+^) to drive the uptake of peptides across the cell membrane [40,41]. The stoichiometry of proton driven peptide transport is currently unknown for the bacterial members of the family. However, evidence from electrophysiological studies on rabbit PepT1 expressed in *Xenopus laevis* oocytes suggests a 2:1 proton to anionic dipeptide stoichiometry [42]. These studies also propose that anionic dipeptides are transported in their neutral and negatively charged forms, with high (μM) and low (mM) affinity respectively. Cationic dipeptides by contrast are transported in neutral and positively charged forms. Interestingly, similar studies on PepT2 suggest a 2:1 and 3:1 proton:peptide stoichiometry for neutral and anionic dipeptides [43]. These results indicate a mechanistically important link between the ionization state of the peptide and the interaction with the transporter, which may have important implications for the design of drugs to utilize these systems for improved uptake.

**Fig. 1:**
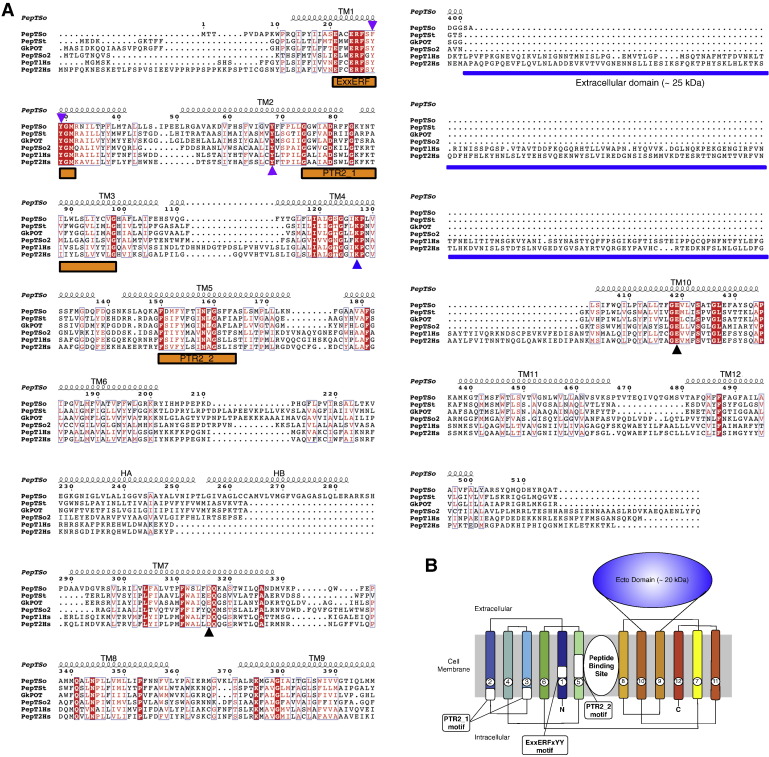
A. Sequence alignment and secondary structure of PepT_So_. Amino acid sequence alignment of *S. oneidensis* PepT_So_ (Uniprot: Q8EKT7), *S. thermophilus* PepT_St_ (Q5M4H8), *G. kaustophilus* GkPOT (Q5KYD1), *S. oneidensis* PepT_So2_ (QHE8ES) with human PepT1 (B2CQT6) and PepT2 (Q16348) homologues using ClustalW. Identical residues are highlighted in red. The α-helices in PepT_So_ are depicted as coils above the sequences. The conserved signature motifs within the PTR family are marked with orange horizontal bars. Residues described in the review are highlighted; proton binding (green triangles), peptide transport (yellow ovals), peptide specificity (gold stars) and extracellular and intracellular gate (blue squares). The horizontal blue bar represents the location of the extracellular domain present in the mammalian homologues but absent in the prokaryotic members. B. Topology diagram of human PepT1 with helices colored blue to red including the position of the extracellular ecto domain between TM9 and 10. The locations of the conserved sequence motifs within the TM helices are shown. Sequence logos (http://weblogo.berkeley.edu/) of these motifs generated from 24 eukaryotic and 19 prokaryotic PTR family sequences aligned as described for human PepT1 and PepT2 are illustrated.

At the primary structure level there exists a remarkably high degree of sequence conservation within the TM helices of the PTR family [6,44] (Fig. 1). Three sequence motifs have been identified, one of which is referred to as the ExxERFxYY motif on TM1. The second two motifs, referred to as the PTR2-1 and PTR2-2 are less well conserved but are nevertheless clearly present throughout the family [6,7,45]. All three motifs are located within the first 180–250 amino acids of PTR transporters and point mutations introduced into these regions typically result in inactive proteins [25,46–50]. Several additional conserved residues have also been shown to affect peptide transport in site directed mutagenesis studies with PepT1 and also cluster within the first six TM helices. A cluster of tyrosine residues (Tyr 64, 91 and 167) play an important role in affecting either the V_max_ or K_M_ of transport [23,25], whereas a conserved tryptophan (Trp294) part of the conserved FWALF motif in TM7, when mutated to an alanine affects both simultaneously [27]. Of the charged residues only one has been identified that is essential for transport, Glu595 in TM10. Within the mammalian POT family there exist four histidine residues, His57, 111, 121 and 261. Of these only His57 is essential for PepT1 function and is predicted to be the site of initial protonation during transport [29]. Many of these residues are present in the bacterial homologues of PepT1 and PepT2, and as discussed below play similarly important roles in proton coupled peptide transport. This conservation between prokaryotic and eukaryotic PTR family members highlights the remarkable degree of the mechanistic conservation within this family of transporters [8].

The first crystal structure of a PTR transporter from the bacterium *Shewanella oneidensis*, PepT_So_, was determined in 2011, revealing a novel occluded conformation for the MFS [40]. This was quickly followed by three more structures, all from different bacterial species. PepT_St_ from *Streptococcus thermophilus* [49], GkPOT from *Geobacillus kaustophilus* [51] and PepT_So2_, a second PTR transporter from *S. oneidensis* [52]. The last two structures were obtained in complex with an antibacterial phosphono di-peptide, alafosfalin, and revealed key structural insights into peptide recognition. We are now uniquely placed to develop a mechanistic understanding of proton coupled peptide transport in this highly conserved and physiologically important transporter family.

## 2 Crystal structures of bacterial members of the PTR family reveal a conserved binding site for peptides

**Fig. 2:**
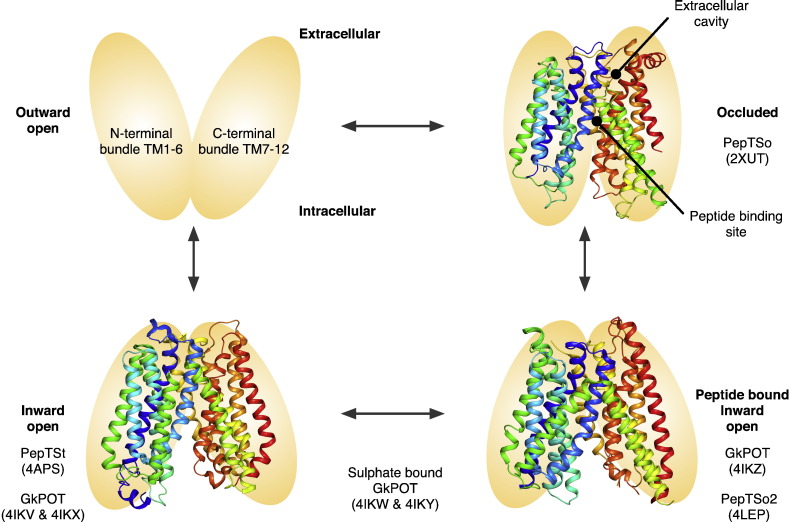
Crystal structures of PTR family transporters. Four crystal structures of PTR/POT family transporters have been determined to date. These represent three unique states in the alternating access transport cycle; ligand bound occluded (PepT_So_), ligand bound inward open (PepT_So2_ & GkPOT) and ligand free inward open (PepT_St_ & GkPOT), shown here in ribbon representation colored from their N-terminus (blue) to C-terminus in their respective states in a simplified model of the alternating access transport cycle. The PDB codes for each structure are given in parentheses.

To date a total of four different PTR family transporters have had their structure determined by X-ray crystallography from different bacterial species (Fig. 2). The four structures represent three unique states of the transport cycle. PepT_So_, the first structure determined, revealed an occluded state, where the central peptide binding site had closed around an unidentified molecule obtained during purification or crystallization (PDB: 2XUT). In contrast, the second structure determined for this family PepT_St_, was captured in the fully open state, with the binding site accessible to the cytoplasmic side of the membrane — a so called inward open state (PDB: 4APS). The third and fourth structures determined were those of GkPOT and PepT_So2_ respectively, and were captured in the inward open state but with a bound peptide sitting in the peptide binding site (PDB: 4IKZ and 4LEP4IKZ4LEP respectively). GkPOT was further crystallized in the inward open apo state and with a sulfate ion bound in the central cavity (PDB: 4IKV, 4IKX, 4IKY & 4IKW4IKY4IKW) and unlike the other proteins was crystallized in the lipidic cubic phase, which allowed diffraction data to be collected at much higher resolution, 1.9 Å for the apo structure and 2.4 Å for the peptide bound complex [51].

The structures revealed the canonical MFS fold with the N- and C-terminal six-helix bundles, formed by TM helices TM1–TM6 and TM7–TM12, coming together in the membrane to form a ‘V’ shaped transporter, related by a pseudo two-fold symmetry axis running perpendicular to the membrane plane [53]. The current POT family structures all have two additional TM helices, which we termed HA and HB that are inserted into the cytoplasmic loop connecting the N- and C-terminal bundles [40]. These form a hairpin in the membrane that packs against the periphery of the protein, although the arrangement is slightly different between the four structures. Their role is currently unclear but these structures do reveal how the canonical 12 TM MFS fold, observed previously for other members of this superfamily [12,13,18,33,37,38], can accommodate additional helices. The absence of these helices in the fungal, plant and mammalian protein sequences however, suggests they do not contribute to a conserved transport mechanism and are specific for the prokaryotic members of the family, perhaps playing a role in stability or folding specific to these species.

The structure of PepT_So_ revealed two hydrophilic cavities. A large central cavity situated within the centre of the membrane and a smaller extracellular cavity located at the interface between the N- and C-terminal domains (Fig. 2) [40]. It is roughly cone-shaped, with the apex at the bottom near the central cavity, opening outward. The overall dimensions of the small cavity are approximately 16 × 8 × 8 Å, too small to accept a peptide though hydrophilic enough to accommodate water molecules. The role of the extracellular facing cavity is currently unclear, but as discussed below it forms as a result of structural flexibility within the C-terminal bundle brought about through the occlusion of an unknown molecule within the central peptide binding site. We currently suspect this cavity may be involved in the transport mechanism, possibly as a route for proton binding to residues within the central peptide-binding site.

**Fig. 3:**
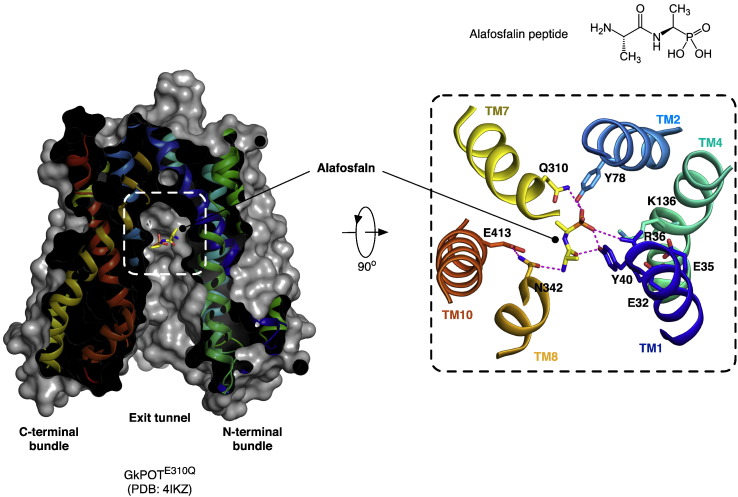
Structure of GkPOT^E310Q^ bound to the phosphonopeptide alafosfalin. A. View through a section of the protein volume in the plane of the membrane showing the central peptide binding site (dashed box) and alafosfalin peptide in sticks. B. Close up view of the binding site rotated 90° to the view in A. Hydrogen bonds between the binding site residues and alafosfalin peptide are shown as magenta dashed lines. The chemical structure of alafosfalin illustrates the similarities and differences to naturally occurring peptides.

The two most recent PTR family structures, from GkPOT and PepT_So2_, respectively, were determined in the presence of an antibacterial phosphono di-peptide, alafosfalin to 2.4 Å and 3.2 Å resolution respectively [51,52]. Alafosfalin had been used previously to study transport in PepT1 and PepT2 and closely resembles a natural peptide, except the carboxy group is replaced by a phosphonate moiety [54]. In both structures the alafosfalin peptide can be seen coordinated within the central peptide binding site observed previously in PepT_So_ and PepT_St_ (Fig. 3). The binding site itself is formed by residues from TM1, TM2, TM4 and TM5 from the N-terminal six-helix bundle and from TM7, TM8, TM10 and TM11 from the C-terminal bundle. Intriguingly however the alafosfalin sits in slightly different positions in the two structures. In GkPOT the alafosfalin peptide sits at the apex of an elongated cone shaped cavity (approximate dimensions 11 Å × 11 Å × 21 Å) that extends out from the centre of the transporter and opens out at the cytoplasmic end of the molecule (Fig. 3A). The complex of alafosfalin with GkPOT was achieved by mutating a key acidic residue, Glu310 on TM7 to glutamine. The authors propose that Glu310 plays a key role in the proton coupling mechanism in GkPOT and its mutation to glutamine is proposed to mimic the protonated state of the transporter [51].

The peptide makes a number of interactions to conserved residues in the peptide binding site; the phosphonate group, which is analogous to the carboxy terminus, forms hydrogen bonds with the side chain of the mutated Glu310, and with the hydroxyl group of Tyr40 and guanidinium group of Arg43 on TM1. As such the peptide makes a direct link between two helices in each of the six TM bundles. Tyr40 forms part of a highly conserved sequence motif, ExxERFxYY on TM1 (Fig. 1). At the opposite end of the peptide the N-terminal amino group is recognized by Asn342 on TM8 and Glu413 on TM10. Glu413 is equivalent to Glu595 in the human PepT1 transporter, and appears to be an essential component of the transport mechanism. Mutation of this residue in any PTR family transporter so far tested results in complete loss of transport [49,51,55], underlining the importance of this acidic side chain in the transport mechanism. The GkPOT structure revealed additional space on the C-terminal side of the peptide molecule, which was speculated may accommodate a third amino acid residue in the case of tri-peptide binding. The arrangement of opposite charges within the binding site may play an important role in the recognition and orientation of peptides. Indeed, a similar arrangement of charges was observed in the periplasmic binding protein OppA from *Salmonella enterica* serovar Typhimurium and DppA from *Escherichia coli* [56,57], where salt bridges to the peptide amino and carboxy termini at either end of the binding cavity act to fix peptides of specific length. Perhaps a similar mechanism operates within the PTR family to preferentially bind di- and tri-peptides as previously suggested [58]. The presence of several possible hydrogen-bond donors and acceptors could also be advantageous in adapting to peptides of various lengths, sequences and charges, as observed in the OppA proteins [59]. Most of the other residues in the binding site are conserved hydrophobic residues, including Ile165, Trp306, Trp440 and Phe441. These residues are likely to provide a suitable environment for peptide side-chains that in general are more hydrophobic than the peptide backbone.

**Fig. 4:**
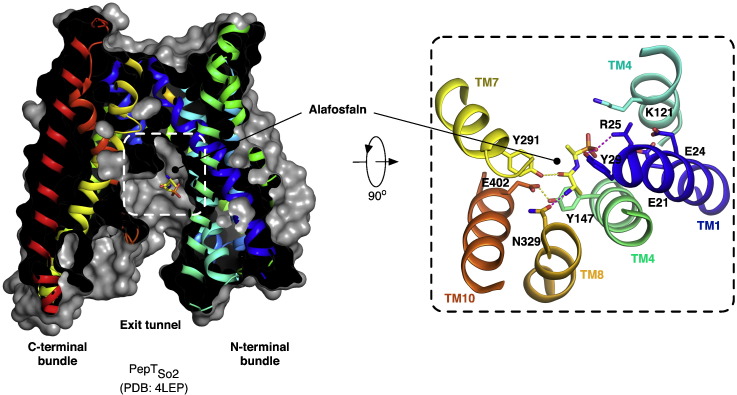
Structure of PepT_So2_ bound to the phosphonopeptide alafosfalin. A. View through a section of the protein volume in the plane of the membrane showing the central peptide binding site (dashed box) and alafosfalin peptide in sticks. B. Close up view of the binding site rotated 90° to the view in A. Hydrogen bonds between the binding site residues and alafosfalin peptide are shown as magenta dashed lines.

By comparison, in the PepT_So2_ structure the position of the alafosfalin is slightly different, with the peptide displaced approximately 3.5 Å towards the cytoplasmic side of the transporter (Fig. 4). Interactions to the binding site residues are still similar to the GkPOT complex, although differences are apparent. The phosphono group is positioned to form a hydrogen bond to Tyr29 and salt bridge to Arg25. These are equivalent to Tyr40 and Arg36 in GkPOT. In PepT_So2_ therefore, the peptide is positioned to interact with the equivalent tyrosine on TM1 but has switched to the arginine in the ExxERFxYY motif. The reason for this appears simple; the equivalent arginine to Arg43 in GkPOT is absent in PepT_So2_, replaced by a glutamine, Gln32. On the opposite side of the binding site, the amino terminus of the peptide sits in a narrow polar pocket formed by Asn151, Asn329 and Glu402. As observed in the GkPOT structure, the amino terminus is recognized through hydrogen bonds to Asn329 and Glu402, which is equivalent to Glu413 in GkPOT and Glu595 in human PepT1, so again we observe further evidence for a role of this glutamate in coordinating the amino terminus of peptides within the binding site of these proteins. Functional data in the form of a thermal stabilization assay was used to confirm the importance of Arg25 and Glu402 in peptide recognition in PepT_So2_, with alanine mutations abolishing the stabilization seen in the presence of di- and tri-peptides in the WT protein [52].

**Fig. 5:**
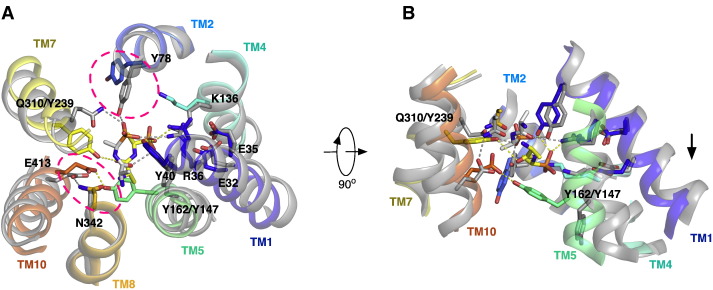
Structural comparison of the alafosfalin binding site between GkPOT^E310Q^ and PepT_So2_. A. Binding site of both GkPOT (colored residues) and PepT_So2_ (gray residues) is shown. The areas that differ most are highlighted by the dashed magenta ovals. B. View rotated 90° with TM8 removed for clarity. The black arrow indicates the displacement of the alafosfalin peptide (~ 2 Å) in GkPOT relative to that in PepT_So2_.

Comparing the two structures reveals a number of interesting observations (Fig. 5). An acidic residue equivalent to Glu300 in GkPOT is found in this region of TM7 in the majority of PTR family members, including the mammalian PepT1 and PepT2 proteins (Fig. 1). Transport studies in both GkPOT and PepT_St_ have identified this residue as playing an important role in coupling peptide transport to the electrochemical proton gradient [49], and where present is essential for transport [49,51,55,60]. In PepT_So2_ however the equivalent residue to Glu300 is a glutamine (Gln290) and the crystal structure reveals no acidic residue is located nearby to cover the role of this residue in proton binding. Instead a tyrosine side chain, Tyr291, sits in a similar place in TM7 and as discussed above is positioned within hydrogen bonding distance to the carbonyl of the alafosfalin peptide (Fig. 4). It is unclear what the mechanistic implications for this are in PepT_So2_, but given the essential role of equivalent acidic residues in this region of TM7 in other PTR family transporters it appears possible that PepT_So2_ may operate using a modified proton coupling mechanism.

Another difference is the position of alafosfalin in the binding site. In PepT_So2_ the peptide is noticeably shifted in favor of interactions with TM1, 7 and 8 and sits ~ 2 Å further down into the cavity than the alafosfalin in GkPOT. In GkPOT additional interactions are observed with TM5, the result of the conserved tyrosine on this helix, Tyr78 moving into the binding site to contact the peptide (highlighted in Fig. 5). In both structures the conserved glutamate on TM10 (Glu413 and Glu402 on GkPOT and PepT_So2_ respectively) have slightly different interaction partners. While in GkPOT Glu413 forms a hydrogen bond to an asparagine, Asn342 on TM8, which in turn contacts the alafosfalin peptide through a hydrogen bond to the amino terminus. In PepT_So2_ the glutamate on TM10, Glu402, interacts with a tyrosine on TM5, Tyr147, linking these two helices together. Interestingly, GkPOT also contains a tyrosine at this position, Tyr162, except in the current structures this side chain adopts a different rotamer position and points away from the peptide-binding site.

The subtle implications of these differences with respect to the mechanism of transport in GkPOT, PepT_So2_ and the wider PTR family are currently difficult to determine. Further biochemical studies will be required to delineate the extent to which these differences represent modifications to a fundamental mechanism of proton coupled peptide transport in these proteins, or whether some members of the PTR family have evolved different mechanisms using similar arrangements of side chains within the binding site.

## 3 Protonation sites in the peptide binding site

PTR family transporters are proton coupled, using the inwardly directed H^+^ electrochemical gradient (Δμ_H_^+^; interior negative or alkaline) [15,61]. As such there must exist specific sites of proton binding that facilitate the uptake and release of peptides. In the GkPOT study the first crystal structure was obtained with a sulfate ion coordinated in the same position as the phosphono moiety of the peptide, i.e. coordinated to Glu310 (Fig. 3) [51]. The 2.0 Å resolution of this crystal structure ensured the accuracy of the observation (PDB: 4IKW). Given the low pKa value for sulfate it was reasonable to suggest the side chain of Glu310 should be protonated in this structure. Supporting this assignment, a further complex of sulfate with the Glu310Gln, which would mimic the protonated state of the glutamate side chain, showed identical interactions. This observation suggests that in the current conformation of the transporter Glu310 has an abnormally high pKa value and is likely to play an important role in the proton coupling mechanism. Various mutants of the equivalent glutamate in PepT1, Glu419 have been reported to drastically reduce transport activity, except where mutation was to an aspartatic acid [60], indicating the importance of a negatively charged residue at this position in the mammalian proteins. Supporting this, mutation of the equivalent glutamate in PepT_St_ also results in loss of transport [49].

**Fig. 6:**
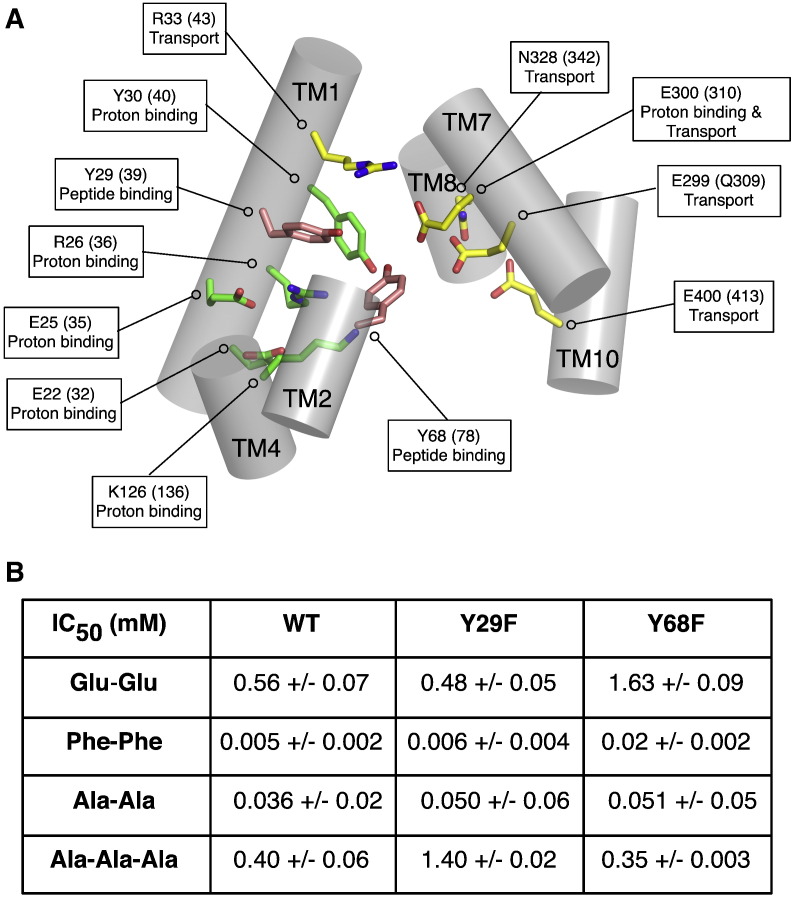
Peptide binding site. A. Zoomed in view of the central cavity in PepT_St_, with the helices represented as cylinders and shown in the plane of the membrane. Side chains observed within the cavity are labeled, with the equivalent residue numbers for GkPOT shown in parenthesis. The function of these residues determined from in vitro assays is indicated. B. Table showing the calculated IC_50_ values for different peptides in the WT and Y29F and Y68F variants of PepT_St_.

Biochemical assays on both PepT_St_ and GkPOT have been reported that also provide additional clues as to the function of conserved side chains in the binding site. Using a combination of proton driven and peptide driven counterflow assays, reconstituted variants of both transporters in liposomes were assayed for their ability to uptake [^3^H]-di alanine [51]. The results of these studies are shown in Fig. 6A for the equivalent residues seen in the binding pocket of PepT_St_. Mutation of Glu22 (32), Glu25 (35), Arg26 (36) or Tyr30 (40) (GkPOT numbering in parentheses) to alanine resulted in transporter variants that could no longer transport under proton driven conditions but could still recognize and transport peptide during counterflow [51]. Within the N-terminal bundle only one residue was found to abolish transport in both assays, Arg33 (43), which as we discuss below is likely to play an important role in regulating the interaction between TM1 and TM7 as part of the extracellular gate and potentially in controlling the protonation state of Glu300 (Glu310). On the C-terminal side of the transporter all the conserved residues assayed produced transport deficient proteins, indicating the C-terminal bundle is more sensitive to mutation.

## 4 Tyrosine residues regulate peptide specificity

The peptide-binding site contains three prominent tyrosine side chains that are conserved within the PTR family (Fig. 1). In PepT_St_ these are Tyr29 and Tyr30 on TM1 and Tyr68 on TM2 (Fig. 6A). Mutation of Tyr30 to phenylalanine resulted in loss of proton driven peptide uptake but retention of counterflow transport, suggesting this residue plays an important role in the proton coupling mechanism [49]. Supporting this role, mutation of Tyr30 to alanine resulted in complete loss of transport in PepT_St_ and GkPOT [51]. In contrast the hydroxyl groups of both Tyr29 and Tyr68 do not contribute to proton binding in PepT_St_, as their mutation to phenylalanine had little impact on proton driven uptake [49].

To investigate the role of Tyr29 and Tyr68 further a series of competition experiments were performed with a library of di- and tri-peptides (Fig. 6B). The phenyalanine mutants showed distinct changes in their affinity for di-Glu and tri-Ala peptides compared to the WT protein, with the alanine mutants losing their ability to transport these peptides altogether while still retaining affinity for di-Phe and di-Ala peptides. This difference suggested Tyr29 and Tyr68 are important in determining peptide specificity, which appears to be supported by the interactions these side chains make to the alafosfalin peptide in the GkPOT and PepT_So2_ structures. To quantify the contribution made by Tyr29 and Tyr68 to peptide affinity, IC_50_ values for these peptides were calculated for each of the phenylalanine mutants. The Tyr29Phe mutant had a decreased affinity for tri-alanine, IC_50_ of 1.4 mM compared with 0.4 mM for the WT, while still retaining WT affinity levels for di-Glu. The Tyr68Phe mutant displays a decreased affinity for di-Glu, IC_50_ values of 1.63 mM compared with 0.56 mM for the WT protein while retaining the same affinity for tri-alanine. Taken together, these results support the importance of the conserved tyrosine residues in peptide recognition, in agreement with previous studies on the mammalian PepT1 transporter [23,62].

## 5 Salt bridge interactions orchestrate communication between the N- and C-terminal bundles during transport

**Fig. 7:**
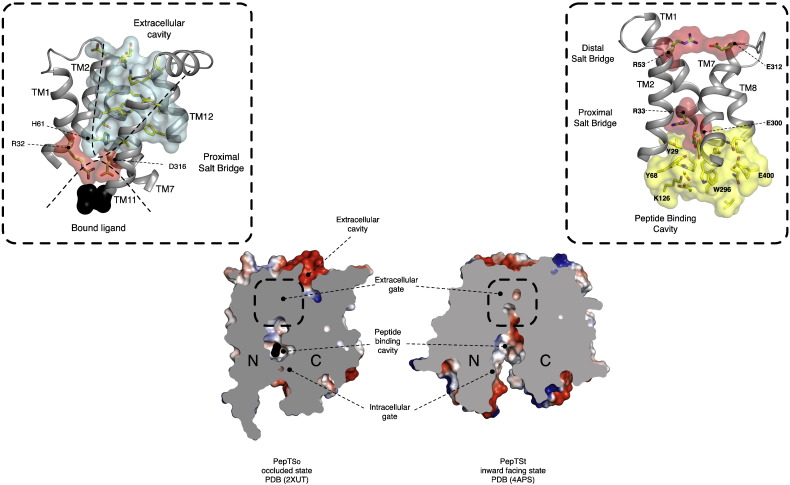
Structural comparison between PepT_So_ and PepT_St_ revealed the nature of the extracellular and intracellular gates. Centre of the figure shows a slice through the volume of PepT_So_ and PepT_St_ positioned as in Fig. 3 & 4 for GkPOT and PepT_So2_ respectively. Key structural regions discussed in the text are illustrated. Top left, zoomed in view of the extracellular gate region in PepT_So_, illustrating the salt bridge interaction between Arg32 on TM1 and D316 on TM7 and their position with respect to the extracellular cavity. Top right, zoomed in view of the equivalent region in PepT_St_, showing the proximal and distal salt bridges stabilizing the interaction between helices TM1, TM2 from the N-terminal and TM7, TM8 from the C-terminal bundles respectively.

To mediate peptide transport across the membrane the PTR transporters must undergo substantial conformational change to enable the central peptide binding site to be alternately exposed to either side of the membrane [45]. These structural changes are often described in terms of the opening and closing of ‘gates’, or localized areas of the protein that either restrict or allow access to the ligand and driving ions to their respective binding sites (reviewed in [46]). In the PTR family the recent crystal structures have allowed identification of the extracellular gate [40,49]. Homology modeling on the outward facing structure of the fucose:proton symporter FucP has further allowed speculation on the nature of the intracellular gate. The extracellular gate is constructed from TM1 and TM2 in the N-terminal bundle and helices TM7 and TM8 in the C-terminal bundle, which in all current PTR structures pack together forming a constriction at the extracellular side of the peptide-binding site. In PepT_St_ these are Arg53 (TM1) with Glu312 (TM7), which forms distal to the central peptide binding site and Arg33 (TM1) with Glu300 (TM7), which forms directly above the binding cavity and which we have called the proximal salt bridge (Fig. 7 right hand side). In GkPOT the equivalent residues are Arg43 (TM1) and Glu310 (TM7) at the proximal salt bridge, although in the alafosfalin complex these are not making a direct interaction due to the presence of the peptide and the interaction of the phosphono group with Glu310. In comparison, PepT_So_ only has the proximal salt bridge formed, between Arg31 (TM1) and Asp316 (TM7), the distal salt bridge being broken, presumably as a result of adopting a more occluded state (Fig. 7 left hand side). Whereas in PepT_So2_ only the distal salt bridge is present, between Asp47 and Arg304. Notice that the charges have swapped in PepT_So2_, which is also observed in GkPOT, where Glu63 (TM1) and Lys322 (TM7) form the distal salt bridge.

In both PepT_St_ mutation of Glu300 to alanine abolished uptake in both a proton driven and peptide driven counterflow assay, whereas transport in the Arg33 mutant was only abolished in the proton driven assay [49,51]. In GkPOT equivalent mutations resulted in similar transport results [49,51]. These results suggest that Arg33 has an important role in facilitating proton coupling but does not affect peptide recognition. In contrast the conserved glutamate on TM7, Glu300 appears essential for peptide recognition, a role also supported by the crystal structure of GkPOT complexed with alafosfalin.

This result suggests a functional subdivision of labor between the two residues at this structurally important region of the transporters. However, for any mechanism to be coupled, one component must rely on the other for function. The result obtained from these assays suggests that these two residues have important complementary roles in proton binding and peptide transport. Clearly the identified salt bridge interactions between Arg33–Glu300 (PepT_St_) and Arg43–Glu310 (GkPOT) participate in both proton binding and peptide transport and facilitate closure of the extracellular gate region. As discussed below, we propose these interactions play an important role in orchestrating alternating access within members of the POT family. However, the structure of PepT_So2_ raises an interesting conundrum, as this apparently important salt bridge interaction is not present, with no obvious alternative substitute (Fig. 4). However, there is an interaction between the equivalent residues, Gln32 (TM1) and Ser325 (TM7), but this is unlikely to operate in the same way as the salt bridge present in the other members of the PTR family.

## 6 A possible induced fit mechanism for peptide transport

**Fig. 8:**
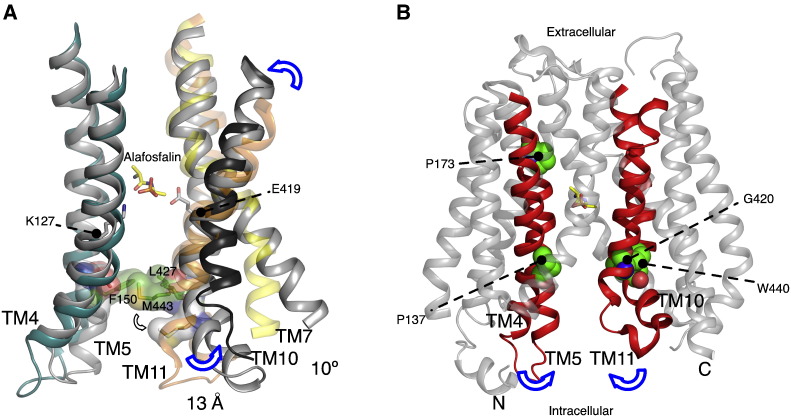
Intracellular gate mechanism. A. Comparison between the inward open PepT_St_ (gray helices) and occluded PepT_So_ structure (colored helices) with arrows showing the hinge like movement that opens the intracellular gate. View is from the membrane plane. Residues forming the intracellular gate are shown as stick models with transparent CPK surfaces. Residue numbers are for PepT_St_. The peptide-binding site containing Lys127 and Glu419 is indicated and alafosfalin modeled in the position occupied in the GkPOT structure. TM11 of PepTSo2 is also shown (dark gray) although in this case the movement of TM11 was more pronounced. B. The occlusion of peptide in the binding site appears to form via an induced fit mechanism with TM4–5 and TM10–11 closing in around the peptide (blue arrows). The four TM helices forming the intracellular gate are colored red. This movement is facilitated by conserved proline residues identified in the MD simulations as being important for the closing of TM4–5 in GkPOT and a conserved glycine and tryptophan residue in TM10 and TM11 respectively that facilitate the reciprocal movement in these helices illustrated in A. The alafosfalin peptide is shown in sticks.

The binding of ligands to proteins often results in structural changes that promote interaction between side chains and the ligand in the binding site. The accumulation of structures from different PTR family members now allows for a useful structural comparison to be made. Prior to the publication of the alafosfalin studies, a comparison between the occluded PepT_So_ structure and inward open PepT_St_ revealed that helices in the C-terminal bundle moved away from their respective positions in the occluded state to open up the central binding site (Fig. 8A) [49]. We observed that this movement was predominantly localized to the cytoplasmic ends of TM10 and TM11, such that the apex of these helices swung away from their symmetrically opposed opposite counterparts, TM4 and TM5. The resulting conformational change caused side chains that had previously blocked exit of the unknown ligand in PepT_So_ (Phe150, Leu427 and Met443) to open up and result in a clear exit pathway from the binding site to the cytoplasm. Structural comparison with PepT_So2_ revealed a similar movement likely occurs in this transporter [52], albeit with a slightly different trajectory with TM11 moving further away than observed in PepT_St_. This movement would also result in the closing of the smaller extracellular cavity observed in the PepT_So_ structure (Fig. 7), and lends support to our earlier hypothesis that helices within the C-terminal bundle are likely to be more dynamic during transport than their N-terminal counterparts.

To analyze the effects of protonation and peptide binding on the dynamics of PTR family transporters, a molecular dynamics (MD) study was conducted on GkPOT in the presence of a POPC lipid bilayer [51]. In 200 ns simulations run in the apo state, the models where Glu310 was protonated remained stable in the bilayer, with rms fluctuations of ~ 1.0 Å between the N- and C-terminal bundles. Interestingly, when Glu310 was deprotonated the same simulation set up resulted in substantial structural changes, with the cytoplasmic half of helices TM4 and TM5 moving in towards TM8 and bending at two conserved prolines, Pro137 (TM4) and Pro173 (TM5). The result of this movement was to occlude a placed di-phenylalanine peptide in the binding site (Fig. 8B). The structural movement observed is very similar to that described above for the C-terminal helices TM10–11, suggesting that during peptide binding the transporter closes up around the peptide through the movement of individual helices, consistent with a recent analysis of other MFS structures [63,64] and resembling an induced fit mechanism of binding. During the simulation, the movement reversed, suggesting that these two states are in equilibrium.

During the structural transition to the partially occluded state, the interaction between Arg43 on TM1 and Glu310 on TM7 in GkPOT is seen to form. This observation suggests that during the simulation Glu310 must lose the proton initially placed at the start of the simulation. Indeed, it was speculated by Doki et al., that the observed interaction of Arg43 might cause the deprotonation of Glu310 through a modulation of the pKa of the side chain [51]. This suggestion certainly seems an appealing way to couple the structural rearrangement of the extracellular gate with the release of a proton from a side chain involved in peptide recognition. However, further studies will be required to confirm this model.

In the MD simulation with the di-phenylalanine peptide, the C-terminal phenyl side chain formed hydrophobic interactions with Tyr78, Trp306 and Trp440 (Fig. 3), appearing to induce the formation of a defined hydrophobic pocket within the binding site [51]. Such hydrophobic pockets have previously been reported for the OppA proteins [65] and help to orientate peptides within the binding surface of these proteins. Similar hydrophobic pockets were also predicted to form within PepT_So_ [40] and the binding site of PepT1. Overall the crystal structures of both the GkPOT sulfate and alafosfalin complexes combined with the MD simulations suggest that protonation and de-protonation of Glu310 may have an important role to play in orchestrating the conformational rearrangements required for transport. The structural comparison with the other PTR family members and the dynamic nature of the molecule in the MD simulations also suggests that the structural rearrangements that orientate the binding site in response to proton and peptide binding are the result of nuanced movements in TMs1, 2, 4, 5 from the N-terminal bundle and TMs7, 8, 10, 11 from the C-terminal bundle.

## 7 A mechanism for proton coupled transport within the PTR family

A large body of experimental data collected on the lactose permease, LacY from *E. coli*, has led to a clearer picture emerging for the role of protons in the sugar:proton symporters [66]. During uphill transport, or the protein working under physiological conditions of lactose accumulation, protonation is required for LacY to bind lactose, whereupon it is the energy of sugar binding and dissociation into the interior of the cell that is predicted to drive the conformational changes that result in transport [67,68]. During release of ligand, sugar dissociates first, causing a conformational change that results in deprotonation into the interior of the cell. In the case of LacY, the close positioning of Arg302 on TM9 to Glu325 on TM10 during lactose transport causes de protonation from the latter side chain [69]; a functional equivalent of which is present in all structures of sugar:proton symporters determined to date [66]. Reorientation of the empty carrier is driven by re protonation from the exterior of the cell.

Given this ordered kinetic scheme, can we make sense of the current experimental data for the PTR family? The proposed de protonation of Glu310 by Arg43 in GkPOT discussed above is similar to that proposed in the sugar:proton symporters. A similar mechanism may therefore exist for the PTR family, where proton binding in the outward facing state precedes peptide binding, whereupon the energy released during peptide binding is used to drive the conformational change that re orientates the binding site during transport. However, in the case of LacY the sugar binding site does not exist in the absence of ligand in the inward open state [70], whereas comparisons between apo and peptide bound GkPOT reveal little structural difference, suggesting the binding site is present in this case [51].

**Fig. 9:**
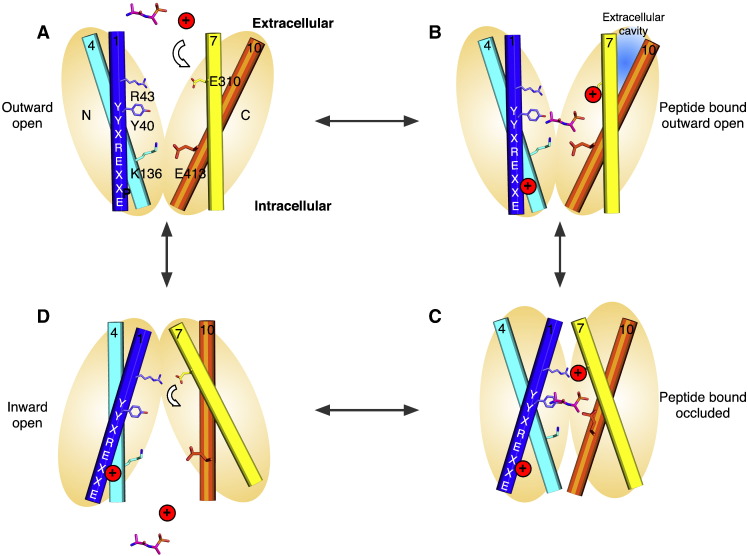
A model for proton driven peptide symport in the PTR family. A. An outward facing state, here modeled on the outward facing fucose permease structure (PDB: 3O7Q) is arbitrarily chosen at the start of the cycle. This state is characterized by the packing of helices TM4–5 with TM10–11 that form the intracellular gate and is potentially stabilized through a salt bridge interaction between K136 and E413 (GkPOT numbering). B. Peptide (here illustrated by the alafosfalin peptide in magenta sticks) and proton H^+^ (red circle) bind from the extracellular side of the membrane. The conserved glutamate on TM7 (E310) is where present likely to play an important role in proton binding, which must facilitate entry of the peptide. Additional important roles in proton binding for the N-terminal ExxERFxYY motif on TM1 and K136 in TM4 are also suggested by the functional data on PepTSt. Conserved tyrosine residues in the binding site play important roles in peptide recognition and specificity. C. Binding results in closure of the extracellular gate to form the occluded state, here modeled on the occluded structure. This conformation is characterized by the packing of helices TM7–8 against TM1–2 at the extracellular side of the binding site, assisted through the formation of the salt bridge interactions between R43 and E310 and by the distal salt bridge between TM2 and TM7 (not shown). Binding of both peptide and proton is also likely to disrupt the proposed interaction between K136 and E413, thereby facilitating release of the intracellular gate. In the occluded state an additional extracellular cavity may also form in the C-terminal domain following closure of the extracellular gate, as observed in the occluded PepT_So_ structure. D. Transition to the inward facing state occurs in part through localized hinge-like movement in helices TM4–5 and TM10–11 that results in release of the intracellular gate, allowing exit of proton and peptide into the interior of the cell. The closing of the extracellular gate and the formation of the salt bridge between R43 and E310 is very likely to result in release of the proton from E310 and this is likely to be coupled to the conformation changes that result in the opening of the intracellular gate helices and ejection of the peptide and proton into the interior of the cell.

A preliminary mechanism for proton coupled peptide uptake within the PTR family is presented in Fig. 9 using side chain numbering from GkPOT (Figs. 1 & 3). In this scheme the outward facing and inward facing states are symmetrical and quite possibly energetically equivalent. Our current understanding of the structures and identification of the gates within the PTR family now reveal that movement between outward and inward open states is very likely composed of many smaller helical rearrangements within functional sub-bundles of helices with each of the N- and C-terminal domains rather than large rigid body movements as previously thought. In the outward open state (Fig. 9A), the intracellular gate is constructed from TM4, TM5 packing against helices TM10, TM11 and stabilized through a putative salt bridge between Lys136 (TM4) and Glu413 (TM10) [49]. The extracellular gate, constructed from TM1, TM2 and TM7, TM8 remain apart and open. Upon binding proton, very possibly to Glu310 on TM7 a di or tri-peptide can enter the binding site (Fig. 9B), whereupon TM1, TM2 and TM7, TM8 begin to close around the peptide (Fig. 9C). This movement is very likely reinforced through the establishment of an electrostatic interaction between the proximal salt bridge between Arg 43 (TM1) and Glu310 (TM7) observed in the crystal structures. This movement must then couple with the weakening and eventual breaking of the intracellular salt bridge, which must result in the peeling away of helices TM4, TM5 from TM10, TM11. This is most likely achieved through an intermediate state similar to the crystal structure of PepT_So_ and resulting in the release of bound proton and peptide into the interior of the cell (Fig. 9D). Particularly interesting with respect to the proposed model is the role of the inverted symmetry repeats within both of the 6-TM bundles [71]. In this model, the first repeat from each bundle contributes to the extracellular gate, whereas the second repeat contributes to the intracellular gate, with two helices acting as support units, TM3, TM6 in the N-terminal bundle and TM9, TM12 in the C-terminal bundle. This produces a blueprint for alternating access within the PTR family that places helices TMs1, 2, 4, 5 and TMs7, 8, 10, 11 in dynamic equilibrium between two presumably energetically equivalent states and TM3, 6, 9 and 12 acting as support rods, as suggested for GlpT, the glycerol-3-phosphate transporter from *E. coli* [71].

## 8 Future perspectives

Recent advances in the structural biology and biochemistry of bacterial PTR family transporters has significantly impacted our understanding of the molecular basis for transport in this family [26]. However, key questions remain to be addressed, particularly with respect to peptide specificity. While information on the role of the conserved tyrosine residues hints at some discrimination, recent evidence from the *E. coli* PTR transporter YjdL suggests that in this case, single amino acids and even tetra-peptides can be transported in a proton dependent manner [72]. Clearly more still remains to be uncovered. Another area of interest concerns the oligomeric state of PTR members. PepT_So2_ was reported to exist as a tetramer in detergent [52], with the suggestion that this higher oligomeric state may have some role in the regulation of transport in vivo. Further studies will be required to address this question for the other PTR members to determine if higher oligomeric complexes are an important part of their physiological state in the membrane. However, with respect to understanding the molecular basis of drug transport through these systems, the most important goal is to tackle the mammalian targets, PepT1 and PepT2. It is highly likely in this author's opinion that important differences will exist in the mechanism of these proteins compared with their bacterial counterparts. An obvious one is the large extracellular domain present between TM9 and TM10 in PepT1 and PepT2 (Fig. 1) that is absent in the bacterial, fungal and plant homologues. Ultimately it will be the determination of their structure and further biochemical investigations that will delineate these differences and reveal the full complexity of this extraordinary transporter family.
